# Assessing the Sustainability of an Integrated Rural Sanitation and Hygiene Approach: A Repeated Cross-Sectional Evaluation in 10 Countries

**DOI:** 10.9745/GHSP-D-21-00564

**Published:** 2022-08-30

**Authors:** Paschal A. Apanga, Matthew C. Freeman, Zoe Sakas, Joshua V. Garn

**Affiliations:** a University of Nevada Reno, Reno, NV, USA.; b Emory University, Atlanta, GA, USA.

## Abstract

**Introduction:**

While many studies have implemented programs to increase sanitation coverage throughout the world, there are limited rigorous studies on the sustainability of these sanitation programs.

**Methods:**

Between 2014 and 2018, the rural Sustainable Sanitation and Hygiene for All (SSH4A) approach was implemented by SNV in sub-Saharan Africa and Asia. Repeated cross-sectional household surveys were administered annually throughout program implementation and 1 to 2 years following completion of program activities. We characterize to what extent sanitation coverage was sustained 1 to 2 years after implementation of this SSH4A intervention.

**Results:**

Surveys were conducted in 12 program areas in 10 countries, with 22,666 households receiving a post-implementation survey. Six of 12 program areas (Bhutan, Ghana, Kenya, both Nepal sites, Tanzania) had similar coverage levels of basic sanitation 1–2 years post-implementation, whereas there were varying levels of slippage in the other program areas (both Ethiopia sites, Indonesia, Mozambique, Uganda, Zambia), ranging from a drop of 63 percentage points in coverage in Ethiopia to a drop of only 4 percentage points in Indonesia. In countries that experienced losses in the coverage of household sanitation, sanitation sharing among neighbors generally did not increase, whereas open defecation did increase. In each of the areas where slippage occurred, the sanitation coverage levels at the final time point were all still higher than the initial time point before SNV started working in these areas. We found several factors to be associated with the sustainability of sanitation coverage, including household socioeconomic status, having household members with disabilities, baseline sanitation coverage levels of the program areas, and rate of change of coverage during program activities.

**Conclusions:**

Data revealed sustained gains in sanitation coverage in some program areas, yet slippage in other areas. This work may serve to benchmark the sustainability of sanitation interventions in sub-Saharan Africa and Asia.

## Abstract (precis)

An evaluation of area-wide sanitation interventions in 10 countries found that 6 of the 12 program areas had sustained similar levels of basic sanitation 1–2 years post-implementation, with varying levels of slippage in the other program areas.

## Teaser

**Key Findings:**

- The Sustainable Sanitation and Hygiene for All (SSH4A) approach was designed to increase sanitation and hygiene coverage with the premise that by working with local government to plan and budget for the intervention, access and uses would be sustained.
- In 6 of the 12 program areas where SSH4A was implemented, we found sustained basic sanitation coverage 1–2 years post-implementation; all program areas, including those with slippage, had higher sanitation coverage than at baseline.
- Community, household, and structural factors were associated with higher toilet sustainability, including higher household socioeconomic status and living in communities with higher baseline sanitation coverage.

**Key Implications:**

- Policy makers, program managers, and funders should continue prioritizing the sustainability of sanitation and hygiene programs. Our findings can help benchmark sanitation sustainability and its predictors in sub-Saharan Africa and Asia.

## INTRODUCTION

The Sustainable Development Goal (SDG) target 6.2 aims to achieve access to adequate and equitable sanitation and hygiene for all and to end open defecation by 2030.^1^^–^^3^ Though it is important for sanitation programs to provide equitable access to sanitation, the sustainability (e.g., preventing stalled progress or slippage of coverage) of sanitation programs is critical^4^^,^^5^ and has been a challenge.^6^

Slippage in sanitation coverage after completion of intervention activities has been a recognized challenge within the sector for many years.^7^ Past interventions focused on toilet access and would typically promote 1 type of toilet option to all households in a village while subsidizing a limited number of toilets for the poorest households.^8^ However, many subsidized toilets were used for other purposes or abandoned, and the approach rarely led to area-wide coverage and toilet use. Community Led Total Sanitation (CLTS)—a demand creation approach that prioritizes collective behavioral change—was developed in response to this lack of behavior change and area-wide sanitation improvements. However, CLTS also had challenges associated with sustainability, driven by the quality of latrines in some areas.^9^ Recent approaches have tried to build on the success of CLTS, while also addressing the supply side limitations. One such example is the Total Sanitation and Sanitation Marketing (TSSM) approach in Southeast Asia that is based on CLTS and complementary social marketing techniques.^10^^–^^12^ The rural Sustainable Sanitation and Hygiene for All (SSH4A) was developed as a response to TSSM, seeking further integration of governance and behavioral change communication (BCC) components in the framework.

Slippage in sanitation coverage after completion of intervention activities has been a recognized challenge within the sector for many years.

The SSH4A approach was developed by SNV and its partners starting in 2008, in partnership with government line agencies, and subsequently tested in 5 countries in Asia.^13^ This approach has been implemented across 18 countries in Africa and Asia and has led to the strengthening of government-run programs. Unlike previous approaches focused primarily on-demand creation, SSH4A is multidimensional in addressing barriers that other sanitation programs may not consider. The SSH4A approach aims to increase sanitation coverage by focusing on strengthening capacity around 4 components: (1) demand creation; (2) strengthening sanitation supply chain and finance; (3) hygiene BCC; and (4) water, sanitation, and hygiene (WASH) governance. SSH4A focuses on capacity building primarily of government line agencies as duty bearers, including creating space for the development of local leadership, tailoring social mobilizing outreach mechanisms, and creating sustainable processes for demand creation and behavior change delivery steered by the local government workers. The approach is integrated with local government planning and budgeting with the intention that activities would be sustained after SNV programs ended.^13^ Extensive country-specific programmatic adaptations and focus points of SSH4A in our specific study setting have been published elsewhere.^14^ Between 2014 and 2018, the SSH4A approach was implemented in 15 countries in sub-Saharan Africa and Asia. Evaluation of data from 11 of these countries revealed notable gains in sanitation coverage across countries.^14^ In 2018, program activities ceased and data were subsequently collected 1 or more years post-implementation.

It is uncommon for sanitation projects to report on post-implementation sustainability.^15^ Many of the studies that have reported on sustainability were CLTS studies. Some of these CLTS studies found returns to open defecation 2–4 years after the completion of CLTS activities.^16^^,^^17^ Other CLTS interventions in Ethiopia and Ghana found open defecation increased by 8 percentage points in the year after the end of intervention activities in 1 of 4 study areas.^9^ Mukherjee et al. evaluated the TSSM program in Indonesia and found that communities that had achieved open-defecation free (ODF) status within 2 months of triggering TSSM activities were more likely to sustain higher coverage of improved sanitation compared to communities that took many months to achieve ODF status.^12^ Two of the 20 communities in this TSSM program returned to open defecation between 4 and 24 months after being declared ODF. As there is a breadth of types of sanitation interventions, there is a need for studies that characterize the sustainability of broader intervention types beyond just CLTS and TSSM.

Because of the breadth of types of sanitation interventions, there is a need for studies that characterize the sustainability of broader intervention types beyond CLTS and TSSM.

In this study, we assessed whether the gains in sanitation coverage made by local governments and stakeholders with support of the SSH4A intervention in 10 countries were sustained 1 to 2 years after the completion of program activities. We characterized the prevalence of basic sanitation access and of various sanitation technologies over time to assess if there was slippage, sustained coverage, or gains in these variables after implementation of the intervention. We also characterized the sustainability of hygiene behaviors, including prevalence of handwashing stations and child feces disposal practices. Finally, we investigated how varying community, household, and structural factors were associated with ongoing sanitation sustainability.

## METHODS

### Study Context

We evaluated whether attained coverage levels of key variables (basic sanitation, safe disposal of child feces, handwashing stations) were sustained after implementation completion. The results from that previous evaluation have been published elsewhere.^14^ A post-implementation visit consisted of cross-sectional household surveys administered in 12 program areas in 10 countries at least 1 year after completion of intervention activities. Study countries included Bhutan, Ethiopia (2 areas), Ghana, Indonesia, Kenya, Mozambique, Nepal (2 areas), Tanzania, Uganda, and Zambia. Program staff were no longer implementing the intervention activities but did return to administer the surveys. The context of the SSH4A approach in each country is briefly described in the following section, and further details have been published elsewhere.^14^

#### Bhutan

At baseline, nearly all households in the Bhutan study area had an unimproved toilet constructed primarily for compliance purposes (i.e., far away from the house and generally not used). SSH4A focused on providing access to improved toilets according to the needs and preferences of households. The preferred option for most households was a flush/pour-flush toilet. Thus, the program had a stronger emphasis on sanitation supply chain strengthening through linkage between potential suppliers and the communities. Large-scale marketing was not feasible due to the remoteness of many of the communities. As a result, local masons were trained on how to build improved latrines and health workers were oriented to critical aspects of toilet construction so they could individually reach out to households and communities.

#### Ethiopia

In Ethiopia, the government implemented the national ONEWASH program that sets the cadre for all (rural) WASH interventions. Within that program, Community Led Total Sanitation and Hygiene was the preferred approach. The SSH4A approach strengthened the existing program by building on-demand creation, while also building awareness and capacity of other aspects, including local sectoral alignment and BCC towards sanitation and hygiene; institutionalizing BCC; revitalizing WASH teams from different sectors to coordinate WASH activities; and establishing and strengthening sanitation marketing centers and artisans to produce and provide sanitation and hygiene products and services to the community at affordable costs.

#### Ghana

Ghana uses the Rural Sanitation Model and Strategy (RSMS), which employs different approaches to promoting access to sanitation and hygiene. The RSMS adopted the CLTS as 1 of the preferred rural sanitation approaches.^9^ The SSH4A approach incorporated BCC into the existing sanitation program in Ghana using several methods to conduct outreach on improving sanitation and handwashing practices. BCC campaigns focused on an “All hands Clean” handwashing campaign as well as maintaining and keeping all latrines clean. The program also emphasized providing a wide range of low-cost sanitation options, including the SAFI latrine (i.e., a product of an action research project conducted by SNV). It also incorporated a multistakeholder approach, including buy-in from local government and traditional authorities (i.e., those who were very instrumental in driving behavior change in target communities), who could then steer demand creation and help scale uptake. Training of government staff and artisans took place to improve entrepreneurial skills, develop capacity, and raise awareness of reliable sanitation options.

#### Indonesia

In Indonesia, the SSH4A approach focused on triggering demand for flush/pour-flush toilets, due to local preference and readily available water in targeted communities. This required strengthening the sanitation supply chain and creating a variety of sanitation product choices. The program anchored its intervention in the national Sanitasi Total Berbasis Masyarakat (STBM), a CLTS approach, to align with national policy guidelines and with the programmatic scope of the relevant line agencies while seeking to increase their attention to issues of quality containment, inclusion for people with different needs, and sustainable use. The buy-in of local elected leaders (Bupatis) was a critical aspect for the long-term institutional integration, financing, and scaling up of the intervention.

#### Kenya

The SSH4A approach in Kenya aimed to create demand for sanitation in alignment with the national government’s CLTS protocols, as well as to stimulate businesspeople to offer affordable toilet and handwashing options through interventions targeting supply chain constraints and product acceptability/accessibility. The program encouraged communities to make informed choices and maintain safe hygiene practices through responsive BCC campaigns based on formative research. Sanitation being a devolved function in Kenya, the program worked with the county governments in area-wide planning, evidence-based strategy adoption, and development of county legislation on sanitation services.

#### Mozambique

The SSH4A approach in Mozambique aimed to develop local government capacity and to build systems for the sustainable delivery of sanitation and hygiene services. Since 2014, the SSH4A approach started early consensus building toward community-led approaches, such as CLTS, as the main entry point for rural sanitation with relevant stakeholders. This built the capacity of local government staff to use BCC and develop sanitation markets to raise community awareness and increase access to affordable sanitation options.^18^ It also included supporting communities in the construction of practicable low-cost sanitation options and conducting BCC campaigns for handwashing with soap.

#### Nepal

The SSH4A approach in Nepal established a strong base for governance by supporting the formation and/or strengthening of multistakeholder WASH coordination committees at the sub-national, district, and local levels. The SNV team adapted CLTS triggering tools, including “political triggering” and post-triggering strategies to motivate the diverse set of communities to invest in their own toilets. The team used BCC campaigns that started soon after triggering. To support the selection of affordable and suitable technologies, ring producers, masons, and hardware suppliers were linked to communities during demand creation activities.

#### Tanzania

In Tanzania, the local government authorities (LGAs) were implementing a National Sanitation Campaign (NSC) aimed at increasing households’ access to improved sanitation. The SSH4A approach focused on strengthening the capacity of the LGAs and local leaders to adopt an integrated approach by combining sanitation demand creation through CLTS with stimulation of business entrepreneurs to offer affordable improved toilet options, including the SAFI latrine. The SAFI latrine was developed by SNV specialists in Tanzania to provide a safe, durable, comfortable, clean, and affordable option for an improved latrine facility. While BCC was used to sustain the use of toilets and handwashing with soap, WASH governance was used to motivate district teams, local leaders, and communities to commit, participate, and contribute to increased access to improved sanitation and hygiene.^19^

#### Uganda

In Uganda, the CLTS approach was already being used^17^ but needed a scaling strategy.^20^ The SSH4A approach emphasized strengthening district water and sanitation governance, community outreach and empowerment, training leaders and stakeholders on toilet quality, engaging with supply chain actors, and disseminating BCC messages, particularly targeting low-income households and households with persons with disabilities. CLTS triggering was complimented with the *Follow Up MANDONA* approach, which was used to motivate communities to adopt behavior change by undertaking simple, immediate, and doable actions to rectify sanitation and hygiene anomalies and drive communities towards ODF status.

#### Zambia

The SSH4A approach in Zambia focused on demand creation, BCC for hygiene, sanitation governance, and supply chain development. SNV trained community champions supported by sanitation action groups in each village to implement CLTS intervention. The Government of Zambia selected CLTS as an approach of choice towards eliminating open defecation through construction of adequate latrines (i.e., smooth cleanable floor, offers privacy, orifice covered by lid, and has handwashing facility). Artisans were trained on production of durable and affordable latrine options to meet the demand of households, including vulnerable households. Sanitation committees established at various levels enabled households to benefit from bulk purchases of sanitation products.

### Data Collection and Sampling Strategy

During this post-implementation visit, data were collected from 22,666 households between September 2019 and April 2021 (Table S1). Data from the post-implementation visits were compared to previous rounds of data collections, which took place between June 2014 and January 2018, or in the case of Ethiopia 2, between May 2017 and December 2019.

The data collection process was standardized across all countries, using Akvo FLOW mobile application software.^21^ Household surveys were used to collect data on household demographic information, WASH access and use, and direct observations of WASH facilities.

As this study used repeated cross sections of randomly selected households, households within each round of data collection are not necessarily the same. Samples were drawn to be representative and comparable to previous data collections. We used the same multistage cluster sampling scheme that was used in the previous study to select a random and representative sample of the program areas.^14^ In the first stage, subdistricts or districts were randomly selected such that their selection probability was proportional to the population size. In the second stage, a random sample of villages/towns was selected from each district/subdistrict proportional to its size. A random sample of households was then chosen from each village/town, and census data were used to produce sampling weights.^14^ Due to insecurity in Uganda, 3 districts were not sampled during the third data collection round, and to ensure that our analyses were done in identical program areas over time, we did not include the third data collection round for Uganda here. Similarly, due to how the funding was set up in Indonesia, no data collection took place there during the second round.

### Ethics Approval

The study authors received deidentified data to perform this study. Research Integrity at the University of Nevada, Reno reviewed the study and determined that it does not require human research protection oversight by the IRB (1378687-1). SNV received approval from each of the individual countries to collect the data. Trained enumerators collected data from adult members of the household. Informed consent and protecting data anonymity were carried out by SNV for every survey.

### Outcome Variables

Our primary outcome of interest was “basic sanitation,” as defined according to the World Health Organization/UNICEF Joint Monitoring Program for Water Supply and Sanitation standard classification system of sanitation technologies.^22^ We also assessed specific sanitation technologies, including: no toilet, flush/pour flush toilet, pit latrine with slab, and pit latrine without slab/hanging latrine. Safe disposal of child feces and access to a handwashing facility with soap were secondary outcome variables. Among households with a child less than 3 years old, we assessed whether they safely disposed of their child's feces. Access to a handwashing facility was defined as the presence of soap and water within 10 paces of a toilet.

### Predictor Variables

We hypothesized that various community, household, and structural factors might be associated with sanitation sustainability at the post-implementation follow-up. Our chosen predictor variables all had biological plausibility of being associated with sanitation sustainability, based on our literature assessment.^23^ We used the same survey questions from previous years of SNV’s evaluations and also restricted to survey questions that were measured consistently between countries. We used community, household, and structural variables from the survey that were both consistently measured across countries and across time.

#### Community Factors

The 2 community-level factors we assessed both related to the history of the previous sanitation interventions in an area. The baseline sanitation coverage of the county/district before the SSH4A intervention started was used as a predictor of post-implementation sustainability and might be a proxy for the overall maturity of the market development, supply chains, or governance systems. We also assessed if the “rate of change” in sanitation coverage from the previously implemented interventions was associated with sustainability. The rate of change variable compares the baseline sanitation coverage levels to the coverage levels during the final round that SNV was working in the area and also might be related to the maturity of supply chains and commitment to sustained government involvement.

#### Household Factors

We included 2 household characteristics: household wealth quintile, and if there were household members with disabilities. Wealth has been associated with sanitation impact and sustainability in many studies. Wealth quintiles of each country were estimated from household assets using the EquityTool developed by the Social Franchising Metrics Working Group (https://www.equitytool.org/development/) as a guide. We compare households in the lowest 2 socioeconomic status (SES) quintiles to those in the upper 3 quintiles.^24^ We used the Washington Group short set of disability questions to construct our disability variable,^25^ which was defined as a household with a household member who reported a lot of difficulty or inability to see, walk or climb steps, or perform self-care such as washing or dressing.

#### Structural Factors

Structural factors included water table depth, soil type, tank pit location, and toilet age. Water table depth was collected using multiple depth categories and later categorized to between 1 and 3 meters versus 3 or more meters. Higher groundwater tables (e.g., 1–3 meters) may be prone to flooding. Soil type was categorized as solid rock/clay versus other soil types. Different soil types vary in durability and may also be used as construction materials in some countries. The type of soil may affect access to sanitation in several ways. Households within areas with coarser soil types have been less likely to have sanitation facilities compared to areas with finer soils,^26^ as coarser soils are less cohesive and facilitate percolation of water at a higher rate, which makes latrine construction more susceptible to flooding and collapse of existing latrines.^27^ Toilet age was categorized as less than 1 year versus 1 year or more and was respondent-reported.

### Analysis

To assess whether the key outcomes were sustained, we compared the program-level prevalence of outcome variables between the final round while SNV was working in the area and 1–2 years post-implementation to see whether there were changes. Results were stratified by each of the 12 program areas. Analyses were also conducted using linear models (i.e., SURVEYREG procedures) to assess the sustainability of different technology types of the sanitation ladder, where the prevalence of various toilet types was compared between the final post-implementation survey and earlier surveys during implementation of the SSH4A approach. We also characterize how latrine sharing changed during the 1 to 2 years following completion of program implementation. For all of the analyses, we accounted for the stratified design and applied sampling weights to ensure representativeness with the program areas.^14^

We also used linear models (i.e., SURVEYREG procedures) to assess whether community, household, and structural factors were associated with a change in sanitation coverage by introducing interaction terms between each of these factors and post-implementation change in sanitation coverage (factor*change in sanitation coverage). Potential effect modifiers in these analyses include soil type, water table depth, toilet age, pit location, households having any person with a disability, household SES, baseline sanitation coverage, and rate of change in sanitation coverage from the previous 4-year study. Each of these variables was assessed individually in an unadjusted model with all of the data aggregated and the only control variable was indicator variables for each of the program areas under study.

Data were cleaned using STATA version 14 SE (StataCorp) and analyzed using SAS version 9.4 (SAS Institute).

## RESULTS

Six of 12 program areas (Bhutan, Ghana, Kenya, Nepal 1, Nepal 2, Tanzania) had sustained coverage of basic sanitation at levels similar to the final round when SNV was still working in the area (Table 1). Nepal 1 did not have a meaningful decrease in coverage (only a 2 percentage point decrease), but this change was marginally statistically significant due to our large sample size. There were varying levels of slippage of sanitation coverage in the other program areas (Ethiopia 1, Ethiopia 2, Indonesia, Mozambique, Uganda, Zambia), ranging from a drop of 63 percentage points in coverage in Ethiopia 1 to more modest drops (e.g., drops from 4 to 21 percentage points) in the other sites (Table 1). Among these 6 program areas that had decreases in coverage, all still had higher sanitation coverage than at baseline^14^ before SNV started working in the area.

**TABLE 1.** Change of Coverage for Outcomes of Interest 1–2 Years Post-Implementation, Shown by Program Area

	**Basic Sanitation Coverage** ^a^		**Safe Disposal of Child Feces**		**HW Station With Soap Prevalence**	
	**Prevalence in Final Round of SSH4A Intervention, % (95% CI)**	**1-year Point Change Post-Implementation, % (95% CI)**	**Prevalence in Final Round of SSH4A Intervention, % (95% CI)**	**1-year Point Change Post-Implementation, % (95% CI)**	**Prevalence in Final Round of SSH4A Intervention, % (95% CI)**	**1-year Point Change Post-Implementation, % (95% CI)**
Bhutan	95 (93, 97)	+2 (−1, 4)	37 (23, 51)	−32 (−46, −18)^b^	64 (59, 69)	−9 (−16, −2)^b^
Ethiopia 1	95 (95, 96)	−63 (−65, −61)^b^	97 (96, 99)	−60 (−64, −56)^b^	26 (24, 28)	−21 (−23, −19)^b^
Ethiopia 2	99 (99, 100)	−14 (−16, −11)^b^	97 (95, 100)	−14 (−21, −8)^b^	20 (18, 23)	−10 (−13, −6)^b^
Ghana	36 (34, 38)	+3 (0, 6)^b^	47 (44, 50)	−5 (−9, −1)^b^	11 (10, 12)	−3 (−4, −1)^b^
Indonesia	95 (94, 96)	−4 (−6, −3)^b^	79 (74, 84)	−45 (−52, −37)^b^	36 (34, 39)	−4 (−7, −1)^b^
Kenya	68 (66, 69)	0 (−3, 2)	69 (66, 72)	−6 (−10, −2)^b^	10 (9, 11)	+1 (−1, 2)
Mozambique	65 (62, 68)	−15 (−20, −11)^b^	73 (66, 80)	−8 (−18, 3)	19 (17, 22)	+4 (0, 7)
Nepal 1	99 (98, 100)	−2 (−3, 0)^b^	94 (91, 98)	−16 (−26, −7)^b^	70 (67, 74)	+3 (−3, 8)
Nepal 2	94 (94, 95)	0 (−1, 2)	69 (66, 73)	+2 (−3, 7)	76 (75, 78)	−21 (−23, −19)^b^
Tanzania	65 (63, 67)	+3 (0, 6)^b^	96 (94, 97)	−6 (−9, −3)^b^	35 (33, 37)	−27 (−29, −25)^b^
Uganda	78 (77, 79)	−21 (−24, −19)^b^	92 (91, 94)	+1 (−1, 3)	4 (3, 4)	0 (−1, 0)
Zambia	91 (90, 92)	−18 (−21, −16)^b^	94 (92, 96)	−12 (−15, −8)^b^	24 (22, 26)	−21 (−23, −19)^b^

Abbreviations: CI, confidence interval; JMP, WHO/UNICEF Joint Monitoring Programme for Water Supply, Sanitation and Hygiene; HW, handwashing; SSH4A, Sustainable Sanitation and Hygiene for All.

a JMP definition.

b Statistically significant *P*<.05.

Among the 6 program areas that had drops in coverage, all still had higher sanitation coverage than at baseline.

**FIGURE 1:**
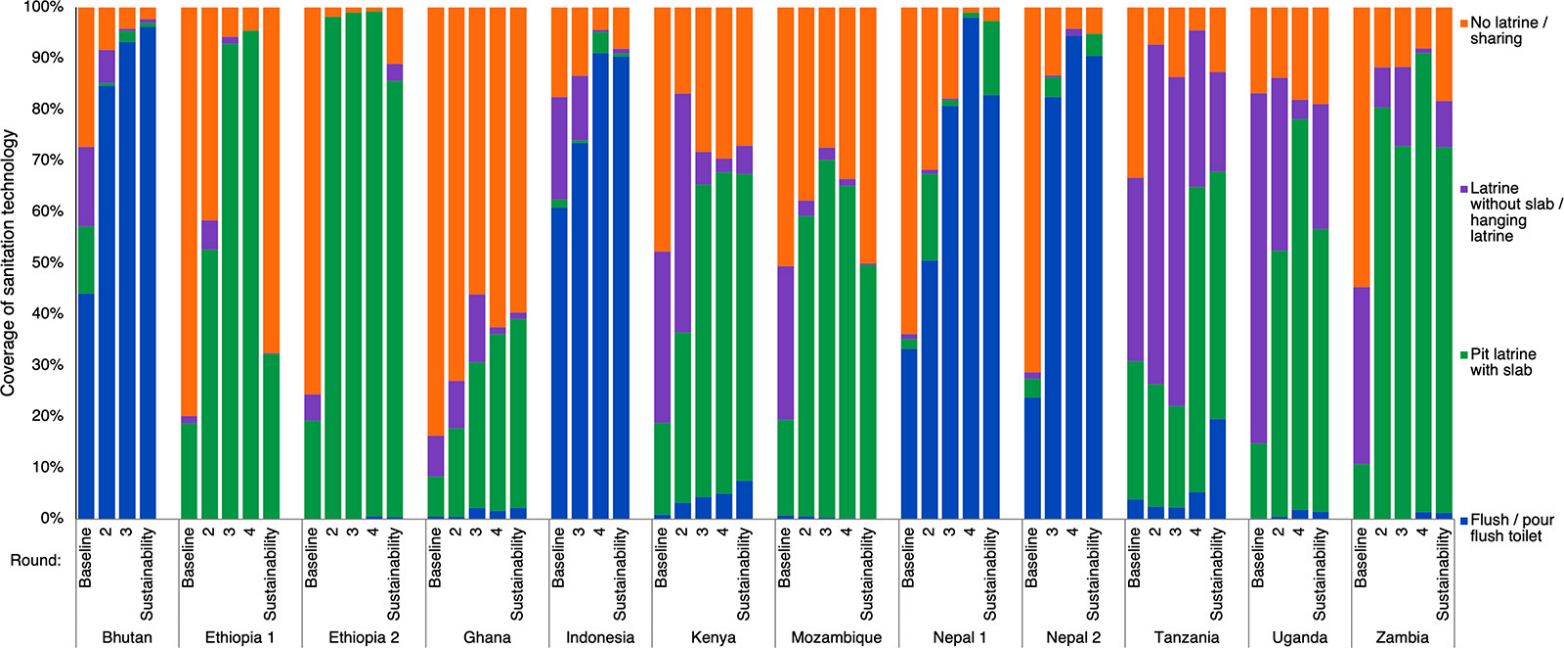
Shift in the Coverage of Various Sanitation Technology Types Across the 5 Rounds

Many of the areas with larger drops in sanitation coverage (e.g., Ethiopia 1 and 2, Mozambique, Uganda, Zambia) were using pit latrines with slabs. However, there was also a drop in flush/pour-flush toilets in Nepal (Figure 1, Table S2). Pour-flush toilets were more common in Asian countries and less common in sub-Saharan African countries. We did a sensitivity analysis using polytomous regression to assess the sustainability of different sanitation technologies (adjusting for SES and country), and we observed that while flush/pour-flush toilets were more likely to be sustained than other types of improved latrines, there was still slippage in the coverage of flush/pour-flush toilets between rounds 4 and 5 (model results not shown).

Nepal 2, and Uganda had no change in the safe disposal of child feces from the final round while SNV was working in the area to the post-implementation round (Table 1). Several program areas had coverage reductions of less than 10 percentage points in safe disposal of child feces (Ghana, Kenya, Tanzania, Mozambique); the other 6 program areas had drops in coverage from 12 to 60 percentage points (Bhutan, Ethiopia 1 and 2, Indonesia, Nepal 1, Zambia). Even though we observed that 9 program areas showed slippage in safe disposal of child feces, 8 of these areas still ended with a higher prevalence of safe disposal of child feces than they had at baseline^14^ before SNV started working in the area.

In 4 of the program areas (Kenya, Mozambique, Nepal 1, and Uganda), there was no difference in the prevalence of handwashing stations with soap at 1–2 years post-implementation (Table 1). The prevalence of handwashing stations with soap dropped in the other 8 program areas (Bhutan, Ethiopia 1, Ethiopia 2, Ghana, Indonesia, Nepal 2, Tanzania, Zambia) between 3 and 27 percentage points. Even though there was slippage of handwashing stations in 8 program areas, all these areas still ended with a higher coverage of handwashing stations with soap than they had at baseline^14^ before SNV started working in the area.

**FIGURE 2:**
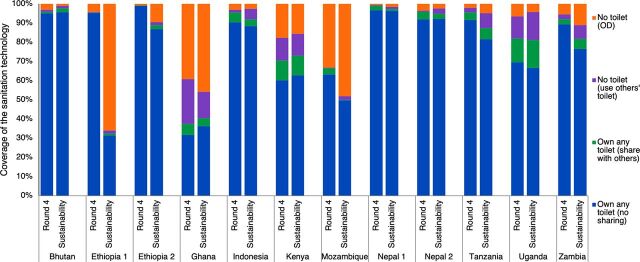
Shift in the Coverage of Any Sanitation Ownership, Shared Sanitation, and Open Defecation, Comparing the Final Round While SNV Was Working in the Area to 1–2 Years Post-Implementation Abbreviations: OD, open defecation.

Trends in coverage of any toilet ownership, sanitation sharing, and open defecation revealed drops in the coverage of any latrine ownership in Ethiopia 1, Ethiopia 2, Mozambique, Tanzania, and Zambia (Figure 2; Table S2). In areas where there were drops in sanitation coverage, sanitation sharing among neighbors did not increase, whereas open defecation usually increased.

Data revealed several community, household, and structural factors associated with sustained sanitation coverage (Table 2). Households in the lowest 2 wealth quintiles were more likely to have had slippage of sanitation coverage (percentage point change=−18%, 95% CI=−19%, −16%) compared to households in the upper 3 wealth quintiles (−11%; 95% CI=−12%, −10%). Households with persons with disabilities were less likely to have had slippage (*P=*.03). In sensitivity analyses assessing households with persons with disabilities and slippage but while also adjusting for potential confounders (e.g., SES and households and the household having an elderly person), the same findings persisted as in the unadjusted model (results not shown). Higher baseline sanitation coverage (i.e., higher sanitation coverage when the intervention started in 2014) was associated with better sustainability compared to having had a low baseline sanitation coverage (*P*<.01). Having had more dramatic improvements in sanitation coverage during the previous 4-year intervention period was also associated with a higher slippage (*P*<.01).

**TABLE 2.** Associations Between Various Community, Household, and Structural Factors and Sustainability of Basic Sanitation^a^ Coverage 1-Year Post-Implementation

**Variable** ^b^	**Post-Implementation Percentage Point Change in Basic Sanitation Coverage, % (95% CI)**	***P* Value**
Household wealth quintiles		
Lowest 2 quintiles	−18 (−19, −16)	<.01
Upper 3 quintiles	−11 (−12, −10)	
HH with any person with disability		
Yes	−8 (−13, −3)	.03
No	−14 (−15, −13)	
Baseline (round 1) sanitation coverage		
75% baseline sanitation coverage	−5 (−8, −3)	<.01
50% baseline sanitation coverage	−9 (−11, −8)	
25% baseline sanitation coverage	−13 (−14, −12)	
Rate of change in sanitation coverage from round 1 to 4 during the previous intervention		
+75% increase in coverage	−22 (−23, −21)	<.01
+50% increase in coverage	−11 (−12, −10)	
+25% increase in coverage	1 (−1, 2)	
Soil type		
Solid rock/clay	−13 (−14, −12)	.26
Other	−14 (−16, −13)	
Water table depth		
1–3 meters	−4 (−6, −2)	<.01
>3 meters	−15 (−16, −14)	
Tank pit above ground level^c^		
Yes	−13 (−16, −11)	<.01
No	−5 (−6, −4)	
Toilet age^c^		
< 1 year	−7 (−10, −5)	.85
1 year or more	−8 (−8, −7)	

Abbreviations: CI, confidence interval; HH, household; JMP, WHO/UNICEF Joint Monitoring Programme for Water Supply, Sanitation and Hygiene.

a JMP definition.

b Each variable represents a separate model that was run. Each model controlled for country program.

c Analysis restricted to those with a toilet. This compares the change in coverage of basic sanitation in the numerator to all types of sanitation in the denominator.

In contrast to our expectations, areas with deeper water tables were more likely to have had declines in sanitation coverage compared to households with shallower water table depths (*P*<.01). In sensitivity analyses to explore this finding, we additionally adjusted for several potential confounding variables (e.g., SES), and the same findings persisted (results not shown). We also performed sensitivity analyses, stratifying to explore if this finding persisted across individual program areas, and the finding appears to be driven by results from a few select program areas (Ghana, Indonesia, Nepal 2, Tanzania), with most program areas showing similar sustainability between deep- and high-water tables (Table S4). There was more slippage among toilets with the tank pit above the ground level compared to toilets with tanks below the ground level (Table 2). There was no difference in slippage from improved sanitation to unimproved when comparing older toilets (≥1 year) and newer toilets (*P*=.85; note, this analysis was restricted to participants who had a toilet).

## DISCUSSION

We assessed the sustainability of sanitation and hygiene coverage in 12 program areas in 10 countries. The multicountry program used the SSH4A, SNV's integrated rural sanitation and hygiene approach. Our analysis found that half of the program areas had sustained statistically similar coverage levels of basic sanitation 1–2 years post-implementation, whereas the other half of the program areas experienced varying degrees of slippage. Among the 6 program areas that had drops in coverage, all still had higher sanitation coverage than at baseline^14^ before SNV started working in the area. This is the first study assessing the sustainability of the SSH4A approach—an approach that has been implemented in 15 countries—and 1 of very few studies characterizing the sustainability of sanitation interventions.

This is the first study assessing sustainability of the SSH4A approach, which has been implemented in 15 countries.

The SSH4A approach was designed within local government planning and budgeting with the hypothesis that the improvements in sanitation and hygiene service delivery systems within program areas would ensure sustainability of access and use post-implementation.^13^ Despite these efforts, some program areas did not fully sustain the intervention achievements. The finding that coverage gains were not sustained in every area is not different from other findings in the literature, as other community-based sanitation interventions have reported returns to open defecation after the intervention activities ended.^12^^,^^16^^,^^17^^,^^28^ Although there were also several program areas where sanitation coverage was fully sustained, it is not fully clear what factors might have led to high sustainability in some areas versus others.

We found a number of community, household, and structural factors to be associated with better toilet sustainability, including households having higher SES, households having members with disabilities, living in communities that had higher baseline sanitation coverage, living in communities that had more gradual previous increases in sanitation coverage, having a tank pit below the ground, and having a toilet in an area with a shallower water table depth. It is not surprising that lower SES households were more likely to have had slippage of sanitation coverage compared to higher SES households. Even while many sanitation programs emphasize reaching lower SES groups, having equitable outcomes by SES often proves difficult. This was also found in the endline measurement of the SSH4A program areas in these countries.^14^ Others have also pointed to poverty as an important factor impacting the sustained use of latrines.^16^^,^^29^^,^^30^ Some of the community, household, and structural factors from the literature^23^ associated with sustained sanitation include seasonality,^5^^,^^29^^,^^31^ infrastructure,^30^ SES,^5^^,^^16^^,^^29^^,^^30^^,^^32^ education,^30^ and household structure.^33^ In contrast to our expectations, areas with deeper water tables were more likely to have had declines in sanitation coverage, but this was primarily driven by 4 program areas (Ghana, Indonesia, Nepal 2, Tanzania), which were all sites where toilet coverage was generally sustained in our overall analyses. Our finding on households with persons with disabilities may be explained by these households having experienced the convenience of a toilet nearby for the care of a member with a disability and subsequently placed a relatively higher priority on maintaining that toilet.

Several studies have reported that factors can lead to increased sustainability, including frequent personal contact with health promoters and accountability over a period of time,^23^ an enabling environment with market access to latrine products,^30^^,^^34^^,^^35^ follow-up monitoring,^30^^,^^35^^–^^37^ social cohesion and social capital among community members,^38^ effective community leadership and political will,^36^^–^^38^ civic pride,^35^ access to sanitation markets and hardware, and sustained behavioral change.^36^^,^^38^ One possible contributor to the high sustainability we observed in many countries may be that the SSH4A approach is an integrated, multidimensional approach with the aim that these different dimensions of the intervention might address the unique sanitation barriers (or enablers) in program areas with different contexts.^14^ While we did not directly assess programmatic factors associated with sustainability, some of the variables we did include may serve as proxies for important programmatic factors. One example is baseline coverage levels. A higher coverage at baseline (before SNV arrived) might be a proxy for the overall maturity of markets, supply chains, and/or governance systems in the area, factors that are likely to be important facilitators/contributors for sustained coverage in some program areas. In areas where overall market development is incipient (e.g., hardware stores are not prevalent), it is more difficult to link and sustain sanitation products in that system. Our findings on steep increases in coverage being associated with higher slippage might also be related to the difficulty of sustaining coverage, sanitation supply chains, and supportive governance practices in a context where overall markets and governance are weak. It may be that in some areas longer timelines and commitment may be required for key stakeholders to develop markets and supply chains, and to maintain the necessary local commitment to enable sustainability.

Higher coverage at baseline might be a proxy for the overall maturity of markets, supply chains, and/or governance systems in the area, which are likely to be important facilitators for sustained coverage in some program areas.

We also observed sustained coverage for some of our secondary outcomes in certain program areas. Safe disposal of child feces and access to handwashing stations with soap were sustained in 3 and 4 program areas, respectively, with varied declines in coverage in the other program areas. Notwithstanding, even in program areas that had slippage in safe disposal of child feces or handwashing stations with soap, there was almost always a higher prevalence of these variables than at baseline before SNV started working in these areas.^14^ Findings of slippage in handwashing stations and safe disposal of child feces have previously been attributed to social norms, water shortages, climatic variability, and socioeconomically disadvantaged households, and these factors have been reported as major constraints to sustained WASH behavior.^5^^,^^39^ Having a functional handwashing station requires a basin, water, and soap, and the primary challenge with sustainability in our study was driven by the lack of soap. This was similar to findings from other studies in Ethiopia, Kenya, Uganda, and Sierra Leone.^17^ Our findings of low sustainability of safe disposal of child feces in many program areas were similar to (or even better than) findings from other sustainability studies. For example, results from a CLTS program in Madagascar showed a decline in the practice of safe disposal of child feces to below baseline levels,^40^ whereas all but 1 of our program areas still ended with a higher prevalence of safe disposal of child feces than they had at baseline.

### Limitations

Our study has several limitations. The data collection periods were not always seasonally aligned, and sanitation and hygiene access may be correlated with seasonality. We also lacked data on some key contextual and programmatic variables that could be of interest to WASH sustainability (e.g., road access). As our data came from 12 program areas in 10 countries, there was considerable heterogeneity between countries, but this also may help make our results generalizable in a variety of contexts. For example, we had several program areas with low-income economies (Ethiopia, Uganda, Mozambique), 1 with an upper middle-income economy (Indonesia), and the remainder had low- to middle-income economies. In addition, our evaluation data are from rural settings and may not be applied to all settings. While the SDGs emphasize equitable access to adequate sanitation and hygiene for all and ending open defecation by 2030,^2^ we were only able to assess equity of sustainability for a small number of our variables (e.g., SES). However, our previous study did address equity of the SSH4A approach.^14^ Due to the aggregation of our outcomes, there is potential for ecological fallacy, where inferences about an individual predictor are based on aggregate data for a group.^41^

## CONCLUSIONS

The literature on sustainability from post-implementation WASH studies is limited, yet these data are critical to policy makers, program managers, and funders to characterize the success rate of programs and potentially benchmark or set targets. There is a need for more rigorous implementation research, including concurrent experimental elements to characterize the programmatic and context-specific elements that might best sustain WASH. While our previous evaluation of the SSH4A approach found significant gains in sanitation and hygiene coverage, and in sanitation specifically,^14^ this sustainability study found that these gains did not always persist after SNV stopped working in a program area. Further rigorous studies are warranted to address how gains in coverage are, or are not, sustained. A more nuanced understanding of the sustainability of various intervention types is important for achieving universal access to adequate and equitable sanitation and hygiene, and the eventual end of open defecation.

## Supplementary Material

GHSP-D-21-00564-supplement.pdf

## Notes

**Peer Reviewed**

**First published online:** July 25, 2022.

## References

[1] Transforming our world: the 2030 Agenda for Sustainable Development. United Nations. Accessed June 27, 2022. https://sustainabledevelopment.un.org/post2015/transformingourworld

[2] Sustainable Development Goals: About the Sustainable Development Goals. United Nations. Accessed June 27, 2022. https://www.un.org/sustainabledevelopment/sustainable-development-goals/

[3] World Health Organization (WHO); UNICEF. *Core Questions and Indicators for Monitoring WASH in Health Care Facilities in the Sustainable Development Goals*. WHO/UNICEF; 2018. Accessed June 27, 2022. https://washdata.org/sites/default/files/documents/reports/2019-04/JMP-2018-core-questions-for-monitoring-WinHCF.pdf

[4] Hutton G Chase C. Hutton G Chase C. The knowledge base for achieving the Sustainable Development Goal targets on water supply, sanitation and hygiene. Int J Environ Res Public Health. 2016;13(6):536. 10.3390/ijerph13060536. 27240389 PMC4923993

[5] Odagiri M Muhammad Z Cronin AA. Enabling factors for sustaining open defecation-free communities in rural Indonesia: a cross-sectional study. Int J Environ Res Public Health. 2017;14(12):1572. 10.3390/ijerph14121572. 29240667 PMC5750990

[6] WaterAid; Plan International; UNICEF. *Guidance on Programming for Rural Sanitation*. Water Aid/Plan International/UNICEF; 2019. Accessed June 27, 2022. https://washmatters.wateraid.org/sites/g/files/jkxoof256/files/guidance-on-programming-for-rural-sanitation.pdf

[7] Abebe TA Tucho GT. Open defecation-free slippage and its associated factors in Ethiopia: a systematic review. Syst Rev. 2020;9(1):252. 10.1186/s13643-020-01511-6. 33143715 PMC7641843

[8] Jenkins MW Sugden S. Human Development Report 2006. Rethinking Sanitation: Lessons and Innovation for Sustainability and Success in the New Millennium. UNDP; 2006. Accessed June 27, 2022. https://hdr.undp.org/system/files/documents//jenkinsandsugdenpdf.pdf

[9] Crocker J Saywell D Bartram J. Sustainability of community-led total sanitation outcomes: evidence from Ethiopia and Ghana. Int J Hyg Environ Health. 2017;220(3):551–557. 10.1016/j.ijheh.2017.02.011. 28522255 PMC5475437

[10] A persuasive plea to become "open defecation free": Indonesia’s Total Sanitation and Sanitation Marketing Program. Center for Global Development. 2015. Accessed June 27, 2022. http://millionssaved.cgdev.org/case-studies/indonesias-total-sanitation-and-sanitation-marketing-program

[11] Mukherjee N Shatifan N. *The CLTS Story in Indonesia: Empowering Communities, Transforming Institutions, Furthering Decentralization*. Institute of Development Studies; 2009. Accessed June 27, 2022. https://www.communityledtotalsanitation.org/resource/clts-story-indonesia-empowering-communities-transforming-institutions-furthering

[12] Mukherjee N Robiarto A Effentrif S Wartono D. *Achieving and Sustaining Open Defecation Free Communities: Learning From East Java*. Water and Sanitation Program; 2012. Accessed June 27, 2022. https://www.wsp.org/sites/wsp/files/publications/WSP_Indonesia_Action_Research_Report.pdf

[13] SNV. *Sustainable Sanitation & Hygiene for All (SSH4A)*. SNV; 2014. Accessed June 27, 2022. https://snv.org/assets/explore/download/ssh4a_factsheet_march_2014_0.pdf

[14] Apanga PA Garn JV Sakas Z Freeman MC. Assessing the impact and equity of an integrated rural sanitation approach: a longitudinal evaluation in 11 sub-Saharan Africa and Asian countries. Int J Environ Res Public Health. 2020;17(5):1808. 10.3390/ijerph17051808. 32164375 PMC7084698

[15] UNICEF. *Evaluation of the WASH Sector Strategy “Community Approaches to Total Sanitation” (CATS) Final Evaluation Report*. UNICEF; 2014. Accessed June 27, 2022. https://www.pseau.org/outils/ouvrages/unicef_evaluation_of_the_wash_sector_strategy_community_approaches_to_total_sanitation_cats_2014.pdf

[16] Hanchett S Krieger L Kahn MH Kullmann C Ahmed R. *Long-Term Sustainability of Improved Sanitation in Rural Bangladesh*. Water and Sanitation Program; 2011. Accessed June 27, 2022. https://www.wsp.org/sites/wsp/files/publications/WSP-Sustainability-Sanitation-Bangladesh-Report.pdf

[17] Tyndale-Biscoe P Bond M Kidd R. *ODF Sustainability Study*. Plan International; 2013. Accessed June 27, 2022. https://www.communityledtotalsanitation.org/sites/communityledtotalsanitation.org/files/Plan_International_ODF_Sustainability_Study.pdf

[18] Water and Sanitation Program (WSP). *Water Supply and Sanitation in Mozambique: Turning Finance Into Services for 2015 and Beyond*. WSP; 2011. Accessed June 27, 2022. https://www.wsp.org/sites/wsp/files/publications/CSO-Mozambique.pdf

[19] SNV. *Tanzania – SSH4A Results Programme First Mid-Term Review Brief*. SNV; 2017.

[20] England P Adyero C Hangi B Jimmy MK Mbaha EP. *Local Governance and Sanitation: Eight Lessons From Community-Led Total Sanitation at Scale Through Local Governments in Uganda*. Water Supply and Sanitation Collaborative Council; 2017. Accessed June 27, 2022. https://www.communityledtotalsanitation.org/sites/communityledtotalsanitation.org/files/Eight_lessons_CLTS_Uganda.pdf

[21] Hartung C Lerer A Anokwa Y Tseng C Brunette W Borriello G. Open Data Kit: tools to build information services for developing regions. In: *Proceedings of the 4th ACM/IEEE International Conference on Information and Communication Technologies and Development*. Association for Computing Machinery; 2010(18):1–12. 10.1145/2369220.2369236

[22] World Health Organization (WHO); UNICEF. *Progress on Drinking Water, Sanitation and Hygiene: 2017 Update and SDG Baselines*. WHO/UNICEF; 2017. Accessed June 27, 2022. https://washdata.org/report/jmp-2017-report-final

[23] Hulland K Martin N Dreibelbis R DeBruicker Valiant J Winch P. *What Factors Affect Sustained Adoption Of Safe Water, Hygiene And Sanitation Technologies? A Systematic Review of Literature*. Evidence for Policy and Practice Information and Co-ordinating Centre; 2015.

[24] Chakraborty NM Fry K Behl R Longfield K. Simplified asset indices to measure wealth and equity in health programs: a reliability and validity analysis using survey data from 16 countries. Glob Health Sci Pract. 2016;4(1):141–154. 10.9745/GHSP-D-15-00384. 27016550 PMC4807755

[25] Washington Group on Disability Statistics. Short set of disability questions. Accessed September 30, 2019. https://www.cdc.gov/nchs/data/washington_group/WG_Short_Measure_on_Disability.pdf

[26] Oswald WE Stewart AE Flanders WD. Prediction of low community sanitation coverage using environmental and sociodemographic factors in Amhara Region, Ethiopia. Am J Trop Med Hyg. 2016;95(3):709–19. 10.4269/ajtmh.15-0895. 27430547 PMC5014283

[27] Legge H Halliday KE Kepha S. Patterns and drivers of household sanitation access and sustainability in Kwale County, Kenya. Environ Sci Technol. 2021;55(9):6052–6064. 10.1021/acs.est.0c05647. 33826310 PMC8154356

[28] Plan Netherlands. Final Evaluation Summary: Empowering Self-Help Sanitation of Rural and Peri-Urban Communities and Schools in Africa. Plan Netherlands; date unknown. Accessed June 27, 2022. https://www.communityledtotalsanitation.org/sites/communityledtotalsanitation.org/files/PlanPanAfrica_Evaluation_Summary.pdf

[29] Whaley L Webster J. The effectiveness and sustainability of two demand-driven sanitation and hygiene approaches in Zimbabwe. J Water Sanit Hyg Dev. 2011;1(1):20–36. 10.2166/washdev.2011.015

[30] Malebo HM Makundi EA Mussa R. *Outcome and Impact Monitoring for Scaling Up Mtumba Sanitation and Hygiene Participatory Approach in Tanzania*. National Institute for Medical Research; 2012. Accessed June 27, 2022. https://web-archive.lshtm.ac.uk/www.shareresearch.org//file/1973/Scaling_up_MTUMBA_report_August_2012.pdf

[31] Simms VM Makalo P Bailey RL Emerson PM. Sustainability and acceptability of latrine provision in The Gambia. Trans R Soc Trop Med Hyg. 2005;99(8):631–637. 10.1016/j.trstmh.2004.10.004. 15927217

[32] Waterkeyn J Cairncross S. Creating demand for sanitation and hygiene through Community Health Clubs: a cost-effective intervention in two districts in Zimbabwe. Soc Sci Med. 2005;61(9):1958–1970. 10.1016/j.socscimed.2005.04.012. 15927329

[33] Barnard S Routray P Majorin F. Impact of Indian Total Sanitation Campaign on latrine coverage and use: a cross-sectional study in Orissa three years following programme implementation. PLoS One. 2013;8(8):e71438. 10.1371/journal.pone.0071438. 23990955 PMC3749227

[34] Qutub SA Salam N Shah K Anjum D. Subsidy and sustainability in urban sanitation: the case of Quetta Katchi Abadis Environment Management Programme. Waterlines. 2008;27(3):205–223. 10.3362/1756-3488.2008.024

[35] Clarke NE Dyer CEF Amaral S Tan G, Vaz Nery S. Improving uptake and sustainability of sanitation interventions in Timor-Leste: a case study. Int J Environ Res Public Health. 2021;18(3):1013. 10.3390/ijerph18031013. 33498840 PMC7908170

[36] Sah S Negussie A. Community led total sanitation (CLTS): addressing the challenges of scale and sustainability in rural Africa. Desalination. 2009;248(1-3):666–672. 10.1016/j.desal.2008.05.117

[37] Tribbe J Zuin V Delaire C Khush R Peletz R. How do rural communities sustain sanitation gains? Qualitative comparative analyses of community-led approaches in Cambodia and Ghana. Sustainability. 2021;13(10):5440. 10.3390/su13105440

[38] Cavill S Chambers R Vernon N. Sustainability and CLTS: Taking Stock. Frontiers of CLTS: Innovations and Insights Issue 4. Institute of Development Studies; 2015. Accessed June 27, 2022. https://opendocs.ids.ac.uk/opendocs/bitstream/handle/20.500.12413/5859/Issue%204%20-%20sustainability.pdf

[39] McMichael C Robinson P. Drivers of sustained hygiene behaviour change: a case study from mid-western Nepal. Soc Sci Med. 08 2016;163:28–36. 10.1016/j.socscimed.2016.06.051 27391250

[40] Water Communications and Knowledge Management (CKM) Project. *Evaluation Report: Madagascar Rural Access To New Opportunities For Health And Prosperity (RANO-HP) Sustainability Evaluation*. ECODIT LLC; 2017. Accessed June 27, 2022. https://www.pseau.org/outils/ouvrages/usaid_madagascar_rural_access_to_new_opportunities_for_health_and_prosperity_rano_hp_sustainability_evaluation_2017.pdf

[41] Schwartz S. The fallacy of the ecological fallacy: the potential misuse of a concept and the consequences. Am J Public Health. 1994;84(5):819–824. 10.2105/AJPH.84.5.819. 8179055 PMC1615039

