# Supplementary material for: Assessing the Sustainability of an Integrated Rural Sanitation and Hygiene Approach: A Repeated Cross-Sectional Evaluation in 10 Countries
Source: Glob Health Sci Pract. 2022 Aug 30;10(4):e2100564. doi: 10.9745/GHSP-D-21-00564 (PMC9426995; doi:10.9745/GHSP-D-21-00564)
Supplement: GHSP-D-21-00564-supplement.pdf [file GHSP-D-21-00564-supplement.pdf]

Supplement to: Garn JV, Apanga PA, Freeman MC, Sakas Z. Assessing the sustainability of an integrated rural sanitation and hygiene approach: a repeated cross-sectional evaluation in 10 countries. *Glob Health Sci Pract*. 2022;10(4):e2100564.
https://doi.org/10.9745/GHSP-D-21-00564

TABLE S1. Sample Size by Each Program Area Stratified by Round of Data Collection

Country	Final round of SSH4A intervention (n=22,145)	Population at the final round of SSH4A intervention	Post-implementation round (n=22,666)	Population at post-implementation round
Bhutan	386	35410	394	35410
Ethiopia 1	2145	531578	2145	531578
Ethiopia 2	753	201078	830	201078
Ghana	2700	512939	2743	512939
Indonesia	1929	174547	1929	174547
Kenya	3071	886178	3083	886178
Mozambique	948	708594	1030	708594
Nepal 1	540	215997	550	215997
Nepal 2	2572	548424	2606	548424
Tanzania	1974	1076487	1987	1076487
Uganda	3499	2277682	3662	2277682
Zambia	1628	673529	1707	673529

Abbreviation: SS4HA, Sustainable Sanitation and Hygiene for All.

Supplement to: Garn JV, Apanga PA, Freeman MC, Sakas Z. Assessing the sustainability of an integrated rural sanitation and hygiene approach: a repeated cross-sectional evaluation in 10 countries. *Glob Health Sci Pract*. 2022;10(4):e2100564. https://doi.org/10.9745/GHSP-D-21-00564

TABLE S2. Shift in Coverage of Various Sanitation Technology Types Across the 5 Rounds

Country	Round	Flush/pour-flush toilet	Pit latrine with slab	Pit without slab /hanging latrine	No latrine /sharing
Bhutan	Baseline	44.09	13.12	15.49	27.30
	2	84.68	0.52	6.49	8.31
	3	93.26	2.07	0.52	4.15
	Sustainability[a]	96.19	0.76	0.76	2.28
Ethiopia 1	Baseline	0.00	18.72	1.40	79.88
	2	0.00	52.70	5.72	41.58
	3	0.00	92.86	1.37	5.77
	4	0.00	95.47	0.00	4.53
	Sustainability[a]	0.00	32.33	0.09	67.58
Ethiopia 2	Baseline	0.00	19.23	5.13	75.64
	2	0.00	98.15	0.00	1.85
	3	0.00	98.99	0.00	1.01
	4	0.53	98.67	0.00	0.80
	Sustainability[a]	0.36	85.30	3.25	11.08
Ghana	Baseline	0.57	7.72	8.05	83.66
	2	0.44	17.33	9.22	73.00
	3	2.17	28.51	13.27	56.05
	4	1.63	34.49	1.36	62.52
	Sustainability[a]	2.23	36.85	1.30	59.61
Indonesia	Baseline	60.91	1.54	20.03	17.52
	3	73.48	0.52	12.60	13.40
	4	91.07	4.10	0.39	4.44
	Sustainability[a]	90.38	0.60	0.95	8.07
Kenya	Baseline	0.85	17.93	33.50	47.71
	2	3.15	33.30	46.67	16.88
	3	4.29	61.07	6.37	28.27
	4	5.03	62.71	2.73	29.54
	Sustainability[a]	7.50	59.97	5.50	27.03
Mozambique	Baseline	0.68	18.72	30.03	50.57
	2	0.52	58.75	2.97	37.76
	3	0.31	69.84	2.44	27.41
	4	0.20	64.89	1.36	33.55
	Sustainability[a]	0.00	49.66	0.29	50.06
Nepal 1	Baseline	33.25	1.91	0.99	63.85
	2	50.53	16.89	0.87	31.70
	3	80.72	1.16	0.30	17.82
	4	98.01	1.00	0.00	1.00
	Sustainability[a]	82.81	14.51	0.00	2.68
Nepal 2	Baseline	23.76	3.67	1.22	71.35
	3	82.45	3.85	0.41	13.29
	4	94.44	0.04	1.32	4.20
	Sustainability[a]	90.53	4.29	0.00	5.19
Tanzania	Baseline	3.84	27.03	35.81	33.32
	2	2.40	23.91	66.42	7.27
	3	2.29	19.84	64.25	13.61
	4	5.27	59.60	30.67	4.46
	Sustainability[a]	19.53	48.37	19.49	12.62
Uganda	Baseline	0.14	14.64	68.41	16.81
	2	0.50	51.93	33.82	13.75
	4	1.83	76.30	3.83	18.03
	Sustainability[a]	1.44	55.21	24.48	18.87
Zambia	Baseline	0.00	10.72	34.60	54.68
	2	0.00	80.46	7.80	11.74
	3	0.12	72.73	15.47	11.68
	4	1.41	89.68	0.86	8.05
	Sustainability[a]	1.21	71.45	9.08	18.26

[a] Indicates when SNV stopped working in the program area.

Supplement to: Garn JV, Apanga PA, Freeman MC, Sakas Z. Assessing the sustainability of an integrated rural sanitation and hygiene approach: a repeated cross-sectional evaluation in 10 countries. *Glob Health Sci Pract*. 2022;10(4):e2100564. https://doi.org/10.9745/GHSP-D-21-00564

TABLE S3. Shift in Coverage of Any Sanitation Ownership, Shared Sanitation, and Open Defecation, Comparing the Final Round While SNV Was Working in the Area to 1–2 Years Post-implementation

Country	Round	Own any toilet (no sharing)	Own any toilet (share it with others)	No toilet (use others' toilet)	No toilet (OD)
Bhutan	Round 4	95.08	0.78	1.04	3.11
	Sustainability	95.69	2.03	1.27	1.02
Ethiopia 1	Round 4	95.19	0.28	0.23	4.3
	Sustainability	31.39	1.03	1.53	66.05
Ethiopia 2	Round 4	98.94	0.27	0	0.8
	Sustainability	86.87	2.05	1.57	9.52
Ghana	Round 4	31.69	5.79	23.26	39.26
	Sustainability	36.22	4.17	13.78	45.84
Indonesia	Round 4	90.37	5.16	1.36	3.12
	Sustainability	88.38	3.55	5.43	2.64
Kenya	Round 4	60.15	10.32	11.79	17.75
	Sustainability	62.69	10.27	11.35	15.69
Mozambique	Round 4	63.11	3.34	0.34	33.21
	Sustainability	49.65	0.3	1.89	48.17
Nepal 1	Round 4	96.64	2.36	0.36	0.63
	Sustainability	96.27	1.05	1.12	1.56
Nepal 2	Round 4	91.95	3.85	0.62	3.58
	Sustainability	92.24	2.58	2.77	2.42
Tanzania	Round 4	91.64	3.9	2.33	2.13
	Sustainability	81.55	5.84	7.73	4.89
Uganda	Round 4	69.56	12.41	11.54	6.49
	Sustainability	66.53	14.59	14.59	4.28
Zambia	Round 4	89.29	2.66	2.57	5.48
	Sustainability	76.49	5.25	7.14	11.12

Abbreviation: OD, open defecation.

Supplement to: Garn JV, Apanga PA, Freeman MC, Sakas Z. Assessing the sustainability of an integrated rural sanitation and hygiene approach: a repeated cross-sectional evaluation in 10 countries. *Glob Health Sci Pract*. 2022;10(4):e2100564. https://doi.org/10.9745/GHSP-D-21-00564

TABLE S4. Unadjusted Association Between Water Table Variable and Sustainability of Basic Sanitation Coverage[a] 1–2 Years Post-implementation, by Country

	**Post-implementation % point change in basic sanitation coverage (95% CI)**		
*Country	Among HHs with shallower water table	Among HHs with deeper water table	P-value
Bhutan	N/A[b]	2% (-1%, 4%)	N/A[b]
Ethiopia 1	-60% (-74%, -47%)	-63% (-65%, -61%)	0.70
Ethiopia 2	N/A[b]	-14% (-17%, -12%)	N/A[b]
Ghana	5% (2%, 8%)	-1% (-6%, 4%)	0.03
Indonesia	1% (-3%, 5%)	-5% (-7%, -3%)	0.01
Kenya	-1% (-8%, 7%)	-1% (-4%, 1%)	0.91
Mozambique	N/A[b]	-15% (-20%, -11%)	N/A[b]
Nepal 1	-2% (-5%, 0%)	-1% (-3%, 1%)	0.46
Nepal 2	9% (6%, 13%)	-2% (-4%, -1%)	<0.01
Tanzania	11% (5%, 17%)	1% (-3%, 4%)	<0.01
Uganda	-25% (-29%, -20%)	-20% (-23%, -18%)	0.11
Zambia	-15% (-33%, 4%)	-18% (-21%, -16%)	0.70

Abbreviations: CI, confidence interval; HH, household; JMP, WHO/UNICEF Joint Monitoring Programme for Water Supply, Sanitation and Hygiene.

[a] JMP definition.

[b] N/A=Not applicable, indicating that countries had a prohibitively small sample size in this stratum (Ns ranging from 0 to 5 households).
